# Effects of living near an urban motorway on the wellbeing of local residents in deprived areas: Natural experimental study

**DOI:** 10.1371/journal.pone.0174882

**Published:** 2017-04-05

**Authors:** Louise Foley, Richard Prins, Fiona Crawford, David Humphreys, Richard Mitchell, Shannon Sahlqvist, Hilary Thomson, David Ogilvie, on behalf of the M74 study team

**Affiliations:** 1 MRC Epidemiology Unit & UKCRC Centre for Diet and Activity Research (CEDAR), School of Clinical Medicine, University of Cambridge, Cambridge, United Kingdom; 2 NHS Greater Glasgow & Clyde and Glasgow Centre for Population Health, Glasgow, United Kingdom; 3 Department of Social Policy and Intervention, University of Oxford, Oxford, United Kingdom; 4 Centre for Research on Environment, Society and Health, Institute of Health and Wellbeing, University of Glasgow, Glasgow, United Kingdom; 5 Centre for Physical Activity and Nutrition Research (C-PAN), School of Exercise and Nutrition Sciences, Deakin University, Melbourne, Victoria, Australia; 6 MRC/CSO Social and Public Health Sciences Unit, University of Glasgow, Glasgow, United Kingdom; Leibniz Institute for Prevention Research and Epidemiology BIPS, GERMANY

## Abstract

**Background:**

Health and wellbeing are partly shaped by the neighbourhood environment. In 2011, an eight kilometre (five mile) extension to the M74 motorway was opened in Glasgow, Scotland, constructed through a predominantly urban, deprived area. We evaluated the effects of the new motorway on wellbeing in local residents.

**Methods:**

This natural experimental study involved a longitudinal cohort (n = 365) and two cross-sectional samples (baseline n = 980; follow-up n = 978) recruited in 2005 and 2013. Adults from one of three study areas—surrounding the new motorway, another existing motorway, or no motorway—completed a postal survey. Within areas, individual measures of motorway proximity were calculated. Wellbeing was assessed with the mental (MCS-8) and physical (PCS-8) components of the SF-8 scale at both time points, and the short Warwick-Edinburgh Mental Well-being Scale (SWEMWBS) at follow-up only.

**Results:**

In multivariable linear regression analyses, cohort participants living nearer to the new M74 motorway experienced significantly reduced mental wellbeing over time (MCS-8: -3.6, 95% CI -6.6 to -0.7) compared to those living further away. In cross-sectional and repeat cross-sectional analyses, an interaction was found whereby participants with a chronic condition living nearer to the established M8 motorway experienced reduced (MCS-8: -3.7, 95% CI -8.3 to 0.9) or poorer (SWEMWBS: -1.1, 95% CI -2.0 to -0.3) mental wellbeing compared to those living further away.

**Conclusions:**

We found some evidence that living near to a new motorway worsened local residents’ wellbeing. In an area with an existing motorway, negative impacts appeared to be concentrated in those with chronic conditions, which may exacerbate existing health inequalities and contribute to poorer health outcomes. Health impacts of this type of urban regeneration intervention should be more fully taken into account in future policy and planning.

## Introduction

The activity, health and wellbeing of individuals and populations are shaped by the social, physical and economic environments in which they live [1–3]. Urban regeneration projects are often touted as improving health and prosperity in deprived populations; however, there is limited evidence to support these claims [4, 5].

Urban regeneration refers to a myriad of activities including housing improvements and broader changes to neighbourhood public spaces [6]. Research indicates that urban regeneration has the potential to improve the wellbeing of local residents [7–9]. However, the evidence is inconclusive, and different aspects of urban regeneration, such as the construction of new motorways, might have different effects. Though motorways may improve mobility, roads and traffic have been shown in cross-sectional research to contribute to noise disturbance and severance (separation of residents from facilities or social networks) in local communities [10–12]. Other studies indicate an association between noise disturbance from traffic [13], or living in industrial areas characterised by noise disturbance and air pollution [14], and poor quality of life or wellbeing. However, there are currently no longitudinal studies examining the long-term effects of motorways on wellbeing in local residents.

Urban regeneration in deprived neighbourhoods may also have implications for health inequalities, as deprivation is itself associated with poorer health and wellbeing [15, 16]. Positive impacts from regeneration in deprived areas might plausibly reduce inequalities at the population level. However, whilst previous regeneration projects have been associated with modest improvements in socioeconomic outcomes, the effects were not larger than corresponding national trends [5]. New major roads may contribute to area-level economic revival, but may also degrade the local environment, contributing to a process of ‘deprivation amplification’ [17] in vulnerable communities and widening existing inequalities.

It is not easy to parse urban regeneration ‘interventions’ into their components and establish causal relationships with behaviour or health, because such interventions are typically both complex and ill-suited to evaluation using randomised study designs. Natural experiments are a burgeoning field of public health research in which exposure to an intervention is not manipulated by the researcher, but is nevertheless used to enable controlled comparisons of outcomes over time [18].

The M74 motorway extension in Glasgow, Scotland was a long-standing dormant transport infrastructure project that was revived in the early 2000s, with the primary aim of reducing traffic congestion on the existing motorway network. The construction of this eight kilometre (five mile), six-lane section of motorway through a predominantly urban, deprived area involved a major change to the landscape. The new motorway was mostly raised above ground, and in addition to the road itself, involved the construction of four motorway junctions, new bridges over existing local roads, the demolition of buildings along the proposed route and the concurrent construction of a new residential development close to one of the new junctions.

This presented an opportunity to examine the activity and health impacts of new transport infrastructure using a natural experimental design. In this study, we aimed to contribute to this evidence base by (a) evaluating the effects of living near an urban motorway on wellbeing in local communities, and (b) exploring potential moderators of this relationship.

## Methods

### Context

Glasgow (593,200 inhabitants) [19] is the fourth largest city in the United Kingdom (UK), has the lowest life-expectancy in the UK [20], and is characterised by extremes of affluence and deprivation [21].

### Design

We conducted a quasi-experimental evaluation of a natural experiment, examining the effects of the M74 motorway extension on the travel and activity patterns, injuries and wellbeing of residents in the local area. The study consisted of a longitudinal cohort within two distinct cross-sectional samples recruited at baseline (2005) and follow-up (2013).

The study was approved by University of Glasgow Ethics Committees (baseline reference FM01304; follow-up reference 400120077). If participants completed and returned a postal survey to the study team, this was taken as implied consent for the data to be used for the purpose of the study. This approach was reviewed and approved by University of Glasgow Ethics Committees at both time points.

Further information on the baseline study hypotheses, methods [22] and sample characteristics [23] can be found elsewhere.

### Study areas

Prior to baseline data collection, three local study areas in Glasgow were defined: an area surrounding the new M74 motorway extension (South); an area surrounding the established M8 motorway, which was built in the 1960s (East); and a control area containing a railway segment but no comparable motorway infrastructure (North) [23]. For a map of the study areas, see Ogilvie 2008 [23]. The areas were iteratively delineated in a Geographic Information System, using spatially referenced census and transport infrastructure data combined with field visits. This process ensured that the study areas had similar overall socioeconomic (e.g. levels of deprivation and unemployment, home and car ownership, and prevalence of chronic illness) and topographical characteristics, but differed in terms of containing a motorway [23]. All areas contained a mixture of residential and other land uses, a mixture of housing stock from traditional high-density tenement housing to new developments, and other major arterial roads.

### Intervention

**Fig 1:**
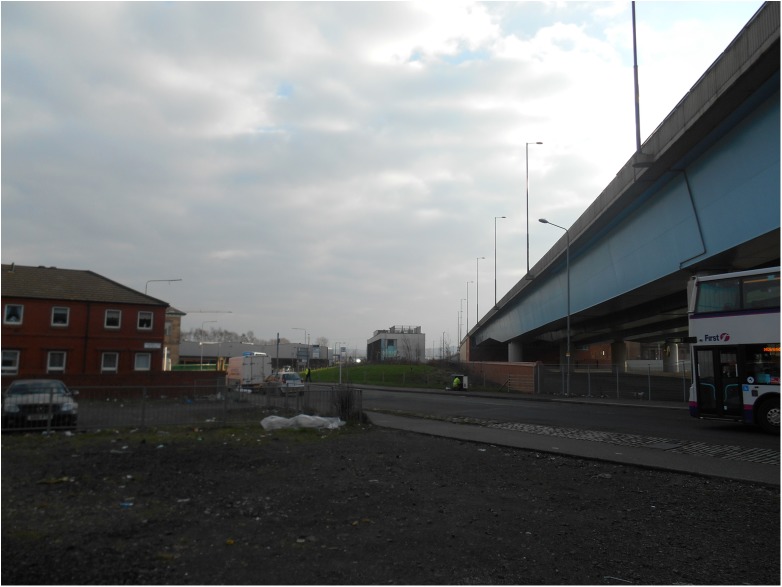
Proximity of housing to M74 motorway extension. Image copyright Amy Nimegeer.

The M74 motorway extension cost approximately £800 million and opened in 2011. The motorway passes through or adjacent to several residential areas, with some homes situated within 50 metres of the carriageway (Fig 1).

Though it was primarily intended to reduce traffic congestion, health-related claims were made about the motorway by supporters within government, and by opponents including members of Scottish parliament, advocacy groups, local businesses and residents. These claims identified potential positive and negative effects of the new motorway on (active) travel, physical activity and wellbeing. At baseline, this dialogue was summarised into two competing overarching ‘hypotheses’: a virtuous cycle in which active travel, physical activity and wellbeing improved, and a vicious cycle in which all declined [22]. At follow-up, these were further developed into a logic model describing the putative causal chains and relationships to be tested *a priori*.

For wellbeing specifically, projected impacts that might worsen wellbeing included loss of green space, visual intrusion, increased traffic noise or vibration, reductions in air quality, severance, the undermining of community facilities and increasing inequalities. Projected benefits that could improve wellbeing included easing traffic on some local roads improving amenity for pedestrians, improved mobility and connectivity to the wider area, and economic regeneration.

### Sampling and recruitment of participants

We recruited participants prior to motorway construction in 2005 (T1), and approximately two years after motorway opening in 2013 (T2). From the three defined study areas, eligible unit postcodes (the smallest unit of postal geography in the UK, corresponding to approximately 15 addresses on average) were identified and a random sample of private residential addresses was drawn from the Royal Mail Postcode Address File. Participants were adults aged 16 years or over who responded to the postal survey delivered to their home address. If more than one householder was eligible, the individual with the most recent birthday completed the survey. At baseline, participants were asked to return an optional consent form giving their permission to be contacted again in the future. Brief contact was maintained via yearly mailings which was intended to promote study retention by providing an ongoing reminder of the study and an opportunity for participants to alert the study team to potential changes in address or circumstance. At follow-up, all baseline participants with current contact details, as well as a new random sample drawn from the Royal Mail Postcode Address File, were mailed a survey.

At baseline, 3,000 surveys were mailed to each study area– 9,000 in total. At follow-up, baseline participants who could still be contacted were accounted for, and the sample for each study area was then topped up to 3,000 with new cross-sectional participants. Therefore, 9,000 surveys in total were mailed at each time point. A minimum achieved sample of 1,200 participants was required at each time point to adequately power the analysis of the primary outcome of the study (travel behaviour).

We followed recommendations to maximise response to postal surveys [24]. Potential participants were sent an initial notification postcard of the survey to come. The next week (the first week of October at both time points), they were mailed a survey and associated study documentation. Those who did not respond were sent the full study documentation a second time approximately one month later. All mailings were staggered over multiple days to maximise the probability that surveys would be completed on different days of the week. Respondents were entered into a £50 prize draw (at baseline) or received a £5 voucher (at follow-up). Responses received more than three months after the first mailing were disregarded, to minimise any effect of seasonal variation in activity patterns.

### Measurement

The survey included items on demographic and socioeconomic characteristics, travel behaviour (including a recall of all travel undertaken on the previous day), physical activity (the short form of the International Physical Activity Questionnaire), health and wellbeing (including the SF-8 scale) and perceptions of the local neighbourhood (S1 Appendix).

#### Wellbeing

Wellbeing was assessed using the SF-8 at both time points, and the short version of the Warwick-Edinburgh Mental Well-being Scale [SWEMWBS] at follow-up only. While some researchers make a distinction between the concepts of health status, (health-related) quality of life and wellbeing, for ease we use the blanket term ‘wellbeing’ here to describe both the SF-8 and the SWEMWBS, acknowledging that these tools do capture somewhat different underlying constructs. The SWEMWBS measures psychological and eudemonic wellbeing, whereas the SF-8 has a dual focus on physical and mental health status, with a specific emphasis on functioning in daily life.

The SF-8 scale is an eight item survey assessing health status in the previous four weeks, derived from (and highly correlated with) the original 36 item version (SF-36) [25]. Items are scored on either 5- or 6-point Likert scales. Using standard procedures, physical and mental component scores (PCS-8 and MCS-8, respectively) were derived [25], whereby higher scores reflect better wellbeing. The SF-8 has been normalised in the general United States population, with mean PCS-8 and MCS-8 scores of 49 [25]. Longitudinal validation in a clinical population has indicated that the SF-8 is sensitive to change, with a clinically meaningful reduction in overall quality of life corresponding to a reduction of 3.0 units for PCS-8 and 3.3 units for MCS-8 [25]. Studies assessing the predictive validity of the original SF-36 scale indicate associations with job loss, use of primary care services, hospitalisation and five-year survival [26].

The SWEMWBS [27] is a seven item survey assessing positive mental wellbeing in the previous two weeks, derived from the original 14 item version (WEMWBS). Items are scored on 5-point Likert scales and summed to produce a total score, whereby higher scores reflect greater wellbeing. Using standard procedures, the raw total score was transformed into a metric score [27]. WEMWBS has acceptable psychometric properties [28] and its mean value in the 2012 Scottish Health Survey was 50 [29].

#### Exposure

In addition to the three study areas, we defined individual-level exposures. Using a GIS, we calculated the distance (metres) from the weighted population centroid of the unit postcode for each participant’s home address in a straight line to the nearest motorway infrastructure. We transformed this exposure using the negative natural log to produce a measure of proximity, whereby higher values reflected greater exposure. Hereafter, we use the term ‘proximity’ to refer specifically to this individual-level exposure.

### Analysis

We explored differences in sample characteristics between study areas and time points, and between the longitudinal cohort and the remainder of the T1 sample, using one-way ANOVA, t and chi-squared tests. We then undertook three main analyses. The first examined within-participant change in the cohort, using SF-8. The second examined population-level change in the repeat cross-sectional sample (in which each participant provided SF-8 data at one time point). The third examined cross-sectional relationships in the full T2 sample, using SWEMWBS.

Linear regression analyses were carried out using Stata13 to assess the relationships of (a) study area and (b) individual-level exposure stratified by study area with (i) PCS-8, (ii) MCS-8 and (iii) SWEMWBS score. The final models were adjusted for age, sex, home ownership, car ownership, working status, perceived financial strain, presence of a chronic condition and years lived in the local area. Additionally, in the longitudinal analysis, we adjusted for the baseline value of the outcome of the model in question. When using study area as the exposure, we used the North area (no motorway) as the reference. For the repeat cross-sectional analyses we added a time point variable, whereby the coefficient of the interaction between time point and motorway exposure gave an indication of the population shift in the outcome over time. We did not impute data as there was less than 5% missing values for all variables.

Finally, we tested all maximally adjusted models for interactions with perceived financial strain and presence of a chronic condition. In models using individual-level exposure, interactions were tested only in the South and East (the areas with a new and an existing motorway, respectively).

## Results

### Response rate

1,345 completed surveys were returned at T1 and 1,343 at T2. After accounting for undeliverable survey packs, the response rate was 16.1% at T1 and 15.8% at T2. 365 participants formed the longitudinal cohort. The remaining 980 (T1) and 978 (T2) participants formed the repeat cross-sectional sample.

### Differences between time points, study areas and samples

Changes in sociodemographic characteristics over time (i.e. age, working status and presence of a chronic condition) were consistent with an ageing cohort. However in the repeat cross-sectional sample, there was a higher proportion of men, car owners and participants with a chronic condition at T2 compared to T1, and the T2 sample was older on average than the T1 sample (Table 1).

**Table 1:** Characteristics of the longitudinal cohort, repeat cross-sectional sample and full T2 sample.

Variable	Longitudinal cohort (n = 365)				Repeat cross-sectional sample (T1 n = 980; T2 n = 978)				Full T2 sample (n = 1343)	
	T1		T2		T1		T2		T2	
	*n*	*mean (SD) / %*	*n*	*mean (SD) / %*	*n*	*mean (SD) / %*	*n*	*mean (SD) / %*	*n*	*mean (SD) / %*
Age (years)	360	50.4 (13.6)	363	58.5 (13.6)**	962	48.8 (18.3)	970	52.6 (16.5)**	1333	54.2 (16.0)
% male	361	43.5	363	44.4	970	37.1	972	42.8**	1335	43.2
% home ownership	360	61.1	363	62.5	965	47.9	971	49.6	1334	53.2
% car ownership	361	58.5	362	60.5	951	48.8	969	53.4**	1331	55.3
% working*	359	58.5	364	48.1**	961	48.3	972	48.3	1336	48.2
% with chronic condition	360	38.9	361	47.9**	955	39.0	964	43.9**	1325	45.0
% perceived financial strain	361		361		955		950		1311	
Quite comfortably off		11.9		12.5		4.9		5.2		7.2
Can manage without difficulty		20.2		24.4		24.0		20.5		21.6
Have to be careful with money		52.9		47.1		51.9		52.4		51.0
Find it a strain to get by		15.0		16.1		19.2		21.9		20.3
Years lived in local area	365	18.3 (15.3)	362	24.9 (16.6)**	980	18.2 (18.0)	965	19.0 (17.4)	1327	20.6 (17.4)
SWEMWBS									1318	21.9 (4.1)
SF-8 PCS-8	352	47.4 (11.0)	360	45.9 (11.7)**	935	46.8 (11.8)	960	45.3 (12.1)**		
SF-8 MCS-8	352	45.5 (11.1)	360	46.4 (11.1)	935	43.8 (11.6)	960	44.4 (12.1)		

n–number; T–time point; SD–standard deviation; SF-8 MCS-8 –SF-8 mental component score; SF-8 PCS-8 –SF-8 physical component score; SWEMWBS–Warwick-Edinburgh Mental Well-being Scale (short version)

*In paid employment (full or part-time), full-time student, or undertaking voluntary work

**Significant difference between time points within the same study sample (p<0.05)

In the longitudinal cohort, there were no significant sociodemographic differences between study areas at either time point. In the repeat cross-sectional sample, there were no significant sociodemographic differences between study areas at T1. However, at T2 on average participants in the North (no motorway) area were older, and participants in the South (new motorway) area perceived less financial strain and had lived in the local area for less time, than those in the other areas. In the T2 cross-sectional sample, participants in the South (new motorway) area perceived significantly less financial strain than those in the other areas (Table 2).

**Table 2:** Sociodemographic characteristics and unadjusted measures of wellbeing by study area and time point.

Variable	Longitudinal cohort (n = 365)				Repeat cross-sectional sample (T1 n = 980; T2 n = 978)				Full T2 sample (n = 1343)	
	T1		T2		T1		T2		T2	
	*n*	*mean (SD)*	*n*	*mean (SD)*	*n*	*mean (SD)*	*n*	*mean (SD)*	n	mean (SD)
Age (years)										
Total	360	50.4 (13.6)	363	58.5 (13.6)	962	48.8 (18.3)	970	52.6 (16.5)**	1333	54.2 (16.0)
North	124	49.0 (13.3)	126	57.3 (13.4)	333	49.7 (18.2)	337	54.6 (16.0)	463	55.3 (15.4)
East	111	51.3 (13.3)	112	59.4 (13.3)	317	48.5 (18.7)	329	51.8 (17.0)	441	53.7 (16.4)
South	125	51.0 (14.1)	125	59.0 (14.1)	312	48.1 (17.8)	304	51.2 (16.4)	429	53.5 (16.1)
% male										
Total	361	43.5	363	44.4	970	37.1	972	42.8	1335	43.2
North	125	37.6	126	38.9	337	36.2	337	43.3	463	42.1
East	111	44.1	112	44.6	318	34.0	331	40.2	443	41.3
South	125	48.8	125	49.6	315	41.3	304	45.1	429	46.4
% home ownership										
Total	360	61.1	363	62.5	965	47.9	971	49.6	1334	53.2
North	125	60.8	126	62.7	337	46.3	336	50.3	462	53.7
East	111	61.3	112	62.5	313	51.1	331	48.6	443	52.1
South	124	61.3	125	62.4	315	46.4	304	50.0	429	53.6
% car ownership										
Total	361	58.5	362	60.5	951	48.8	969	53.4	1331	55.3
North	125	61.6	126	65.9	332	49.4	336	54.8	462	57.8
East	111	52.3	112	55.4	312	49.4	329	52.3	441	53.1
South	125	60.8	124	59.7	307	47.6	304	53.0	428	54.9
% working*										
Total	359	58.5	364	48.1	961	48.3	972	48.3	1336	48.2
North	125	60.8	127	50.4	333	47.2	338	44.4	465	46.0
East	110	54.6	112	46.4	315	48.9	330	49.7	442	48.9
South	124	59.7	125	47.2	313	48.9	304	51.0	429	49.9
% with chronic condition										
Total	360	38.9	361	47.9	955	39.0	964	43.9	1325	45.0
North	126	34.9	125	49.6	329	38.0	334	45.8	459	46.8
East	110	45.5	112	52.7	310	41.0	329	44.1	441	46.3
South	124	37.1	124	41.9	316	38.0	301	41.5	425	41.7
% perceived financial strain										
Total	361		361		955		950		1311	
Quite comfortably off		11.9		12.5		4.9		5.2**		7.2**
Can manage without difficulty		20.2		24.4		24.0		20.5		21.6
Have to be careful with money		52.9		47.1		51.9		52.4		51.0
Find it a strain to get by		15.0		16.1		19.2		21.9		20.3
North	125		126		328		332		458	
Quite comfortably off		12.8		11.1		5.2		3.6		5.7
Can manage without difficulty		23.2		27.8		20.4		20.5		22.5
Have to be careful with money		47.2		42.1		54.9		57.5		53.3
Find it a strain to get by		16.8		19.1		19.5		18.4		18.6
East	110		111		315		322		433	
Quite comfortably off		9.1		9.0		3.8		4.0		5.3
Can manage without difficulty		13.6		21.6		25.7		18.9		19.6
Have to be careful with money		59.1		52.3		51.4		52.5		52.4
Find it a strain to get by		18.2		17.1		19.1		24.5		22.6
South	126		124		312		296		420	
Quite comfortably off		13.5		16.9		5.8		8.1		10.7
Can manage without difficulty		23.0		23.4		26.0		22.3		22.6
Have to be careful with money		53.2		47.6		49.4		46.6		46.9
Find it a strain to get by		10.3		12.1		18.9		23.0		19.8
Years lived in local area										
Total	365	18.3 (15.3)	362	24.9 (16.6)	980	18.2 (18.0)	965	19.0 (17.4)**	1327	20.6 (17.4)
North	127	16.9 (13.1)	126	22.7 (14.1)	338	18.9 (18.7)	332	19.7 (16.9)	458	20.5 (16.2)
East	112	17.5 (13.5)	110	24.9 (14.0)	319	18.2 (16.9)	330	20.7 (18.1)	440	21.7 (17.2)
South	126	20.3 (18.4)	126	27.0 (20.3)	323	17.3 (18.4)	303	16.3 (17.1)	429	19.5 (18.7)
SWEMWBS										
Total									1318	21.9 (4.1)
North									456	21.9 (4.0)
East									439	21.8 (4.1)
South									423	22.0 (4.1)
SF-8 PCS-8										
Total	352	47.4 (11.0)	360	45.9 (11.7)	935	46.8 (11.8)	960	45.3 (12.1)		
North	125	47.5 (10.8)	126	46.2 (11.2)	323	46.7 (11.7)	333	44.9 (12.5)		
East	105	46.7 (11.1)	111	44.7 (12.0)	307	46.7 (11.6)	327	45.0 (11.9)		
South	122	47.7 (11.2)	123	46.7 (11.9)	305	47.0 (12.1)	300	46.2 (12.1)		
SF-8 MCS-8										
Total	352	45.5 (11.1)	360	46.4 (11.1)	935	43.8 (11.6)	960	44.4 (12.1)		
North	125	45.2 (11.6)	126	45.7 (11.9)	323	44.3 (11.6)	333	45.1 (11.7)		
East	105	44.7 (10.6)	111	46.4 (9.7)	307	43.2 (11.7)	327	44.0 (12.7)		
South	122	46.4 (11.1)	123	47.1 (11.5)	305	43.9 (11.6)	300	44.1 (11.8)		

n–number; T–time point; SD–standard deviation; SF-8 MCS-8 –SF-8 mental component score; SF-8 PCS-8 –SF-8 physical component score; SWEMWBS–Warwick-Edinburgh Mental Well-being Scale (short version). North–study area containing no motorway infrastructure; East–study area containing existing M8 motorway; South–study area containing new M74 motorway

*In paid employment (full or part-time), full-time student, or undertaking voluntary work

**Significant difference between study areas within the same time point and study sample (p<0.05)

Compared to the rest of the T1 sample, cohort participants were significantly more likely to be men, to own a home or a car, to be employed or studying, and to describe themselves as financially “comfortably off”, though there were no differences for age, time lived in the local area or presence of a chronic condition.

### Longitudinal analysis of SF-8

There were no significant differences in wellbeing between study areas. In the East (existing motorway) and South (new motorway) areas, participants living closer to a motorway experienced reduced mental wellbeing (MCS-8) over time compared to those further away. In the South, this remained statistically significant in the maximally adjusted model (-3.6, 95% confidence interval [CI] -6.6 to -0.7) (Table 3).

**Table 3:** Longitudinal associations between exposure to a motorway and change in SF-8 physical and mental component score.

	Beta coefficient (95% CI)							
**Exposure**	**Outcome**: SF-8 physical component score							
	n	Model 1	n	Model 2	n	Model 3	n	Model 4
**Area**: East (reference: North)	348	-0.8 (-3.1, 1.5)	346	-0.7 (-2.9, 1.6)	336	-1.1 (-3.4, 1.2)	336	-0.7 (-2.9, 1.4)
**Proximity** within East study area	103	-0.3 (-2.8, 2.3)	103	0.1 (-2.6, 2.7)	100	0.4 (-2.4, 3.2)	100	0.0 (-2.6, 2.6)
**Area**: South (reference: North)	348	0.0 (-2.2, 2.2)	346	0.4 (-1.8, 2.6)	336	0.5 (-1.8, 2.8)	336	0.5 (-1.6, 2.6)
**Proximity** within South study area	116	-0.9 (-3.7, 1.9)	115	-0.4 (-3.2, 2.5)	110	-0.9 (-4.0, 2.3)	110	-0.5 (-3.3, 2.4)
	**Outcome**: SF-8 mental component score							
	n	Model 1	n	Model 2	n	Model 3	n	Model 4
**Area**: East (reference: North)	348	0.8 (-1.9, 3.5)	346	0.7 (-2.0, 3.4)	336	0.5 (-2.2, 3.2)	336	0.8 (-1.6, 3.1)
**Proximity** within East study area	103	-3.5 (-6.7, -0.3)*	103	-2.9 (-6.2, 0.5)	100	-1.2 (-4.6, 2.2)	100	0.2 (-2.5, 2.9)
**Area**: South (reference: North)	348	-0.1 (-2.7, 2.5)	346	0.0 (-2.6, 2.6)	336	0.3 (-2.3, 2.9)	336	0.7 (-1.6, 3.0)
**Proximity** within South study area	116	-3.2 (-6.4, -0.1)*	115	-3.6 (-6.8, -0.5)*	110	-3.9 (-7.2, -0.6)*	110	-3.6 (-6.6, -0.7)*

CI–confidence interval; n–number

*p<0.05

**p<0.01

***p<0.001

Model 1 is unadjusted. Model 2 is adjusted for age and sex. Model 3 is adjusted for variables in model 2 plus home ownership, car ownership, working status, perceived financial strain, presence of a chronic condition and years lived in the local area. Model 4 is adjusted for variables in model 3 plus baseline value of the outcome of the model in question. North–study area containing no motorway infrastructure; East–study area containing existing M8 motorway; South–study area containing new M74 motorway. Proximity refers to the distance from each participant’s home address in a straight line to the nearest motorway infrastructure

### Repeat cross-sectional analysis of SF-8

There were no significant differences in wellbeing between study areas. In the South (new motorway) area, physical wellbeing (PCS-8) reduced over time in people living closer to the motorway compared to those living further away, but this was not statistically significant in the maximally adjusted model (Table 4). In the East (existing motorway) area, a borderline significant (p = 0.06) interaction with chronic condition was found for mental wellbeing (MCS-8). Stratified analysis suggested a reduction in MCS-8 over time among participants with a chronic condition living closer to a motorway compared to those further away (-3.7, 95% CI -8.3 to 0.9).

**Table 4:** Repeat cross-sectional associations between exposure to a motorway and change in SF-8 physical and mental component score.

	Beta coefficient (95% CI)					
**Exposure**	**Outcome**: SF-8 physical component score					
	obs	Model 1	obs	Model 2	obs	Model 3
**Area**: East (reference: North)	1895	0.1 (-2.6, 2.7)	1870	-0.5 (-2.9, 1.9)	1778	-0.8 (-2.6, 1.0)
**Proximity** within East study area	634	2.1 (-1.0, 5.2)	628	0.7 (-2.0, 3.4)	591	1.5 (-0.7, 3.6)
**Area**: South (reference: North)	1895	1.0 (-1.7, 3.6)	1870	0.2 (-2.2, 2.6)	1778	-0.2 (-2.0, 1.7)
**Proximity** within South study area	604	-6.0 (-10.6, -1.5)*	593	-5.2 (-9.4, -0.9)*	571	-1.5 (-4.8, 1.7)
	**Outcome**: SF-8 mental component score					
	obs	Model 1	obs	Model 2	obs	Model 3
**Area**: East (reference: North)	1895	0.0 (-2.6, 2.6)	1870	-0.1 (-2.7, 2.5)	1778	0.5 (-1.8, 2.8)
**Proximity** within East study area	634	-1.3 (-4.5, 1.9)	628	-1.6 (-4.9, 1.6)	591	-0.7 (3.5, 2.1)
**Area**: South (reference: North)	1895	-0.6 (-3.2, 2.0)	1870	-0.6 (-3.3, 2.0)	1778	-0.8 (-3.1, 1.5)
**Proximity** within South study area	604	-3.3 (-7.8, 1.1)	593	-3.7 (-8.2, 0.8)	571	1.4 (-2.6, 5.4)

CI–confidence interval; obs–observations

*p<0.05

**p<0.01

***p<0.001

Model 1 is unadjusted. Model 2 is adjusted for age and sex. Model 3 is adjusted for variables in model 2 plus home ownership, car ownership, working status, perceived financial strain, presence of a chronic condition and years lived in the local area. North–study area containing no motorway infrastructure; East–study area containing existing M8 motorway; South–study area containing new M74 motorway. Proximity refers to the distance from each participant’s home address in a straight line to the nearest motorway infrastructure

### T2 cross-sectional analysis of SWEMWBS

There were no significant differences in wellbeing between study areas. In the East (existing motorway) and South (new motorway) areas, participants living closer to a motorway had poorer wellbeing than those living further away; however, these findings were not statistically significant in the maximally adjusted models (Table 5). A significant interaction with chronic condition was found in the East. Stratified analysis indicated that participants with a chronic condition living closer to a motorway had significantly poorer wellbeing than those living further away (-1.1, 95% CI -2.0 to -0.3).

**Table 5:** Cross-sectional associations between exposure to a motorway and Warwick-Edinburgh Mental Well-being Scale (short version) score at T2.

	Beta coefficient (95% CI)					
**Exposure**	**Outcome**: SWEMWBS score					
	n	Model 1	n	Model 2	n	Model 3
**Area**: East (reference: North)	1318	-0.2 (-0.7, 0.4)	1310	-0.2 (-0.7, 0.4)	1253	0.0 (-0.5, 0.5)
**Proximity** within East study area	437	-0.8 (-1.4, -0.1)*	433	-0.8 (-1.4, -0.1)*	411	-0.4 (-1.0, 0.2)
**Area**: South (reference: North)	1318	0.1 (-0.4, 0.7)	1310	0.1 (-0.4, 0.7)	1253	0.0 (-0.5, 0.5)
**Proximity** within South study area	419	-1.0 (-1.8, -0.2)*	418	-1.0 (-1.8, -0.2)*	404	-0.1 (-0.9, 0.7)

CI–confidence interval; n–number; SWEMWBS–Warwick-Edinburgh Mental Well-being Scale (short version)

*p<0.05

**p<0.01

***p<0.001

Model 1 is unadjusted. Model 2 is adjusted for age and sex. Model 3 is adjusted for variables in model 2 plus home ownership, car ownership, working status, perceived financial strain, presence of a chronic condition and years lived in the local area. North–study area containing no motorway infrastructure; East–study area containing existing M8 motorway; South–study area containing new M74 motorway. Proximity refers to the distance from each participant’s home address in a straight line to the nearest motorway infrastructure.

## Discussion

### Main findings

We found some evidence that living near to either a newly-constructed or an existing urban motorway had a negative impact on local residents’ mental wellbeing. In addition, we found no evidence to suggest any positive effects of living near a motorway on wellbeing.The pattern of findings across the South (new motorway) and East (existing motorway) study areas indicate how adaptation might occur in the short and long term. The negative impacts on wellbeing appeared to be broadly distributed in the short term, becoming concentrated in those with poorer health in the long term.

### Strengths and limitations

This is one of very few intervention studies examining how changes in the environment influence changes in health, particularly in deprived populations. In accordance with calls for more evidence of this nature [18, 30], we objectively defined exposure using multiple methods, used two extensively validated tools to capture the nuances of the wellbeing construct, accounted for a series of potential confounders and used both longitudinal and repeat cross-sectional analyses to offset the limitations of each approach and corroborate findings.

We also acknowledge the limitations of our study. There was relatively high attrition of the longitudinal cohort, though the rate was comparable to that of other similar studies [8, 31] and the repeat cross-sectional design was chosen to buffer against this specific weakness. We also found some differences between study areas for sociodemographic variables at follow-up, despite having delimited comparable study areas and recruited comparable samples at baseline [23]. A natural experimental study design cannot eliminate the possibility of unmeasured confounding related to other concurrent changes, such as the ongoing Clyde Gateway initiative (a regeneration project incorporating parts of the South study area) and the 2014 Commonwealth Games. Additionally, the findings are likely to be at least somewhat specific to the context.

### Comparison with other studies

The reduction in MCS-8 attributable to motorway exposure was approximately 3.5 units in both the longitudinal analysis and the stratified repeat cross-sectional analysis. With the log transformation, this represents the average difference between those living approximately 100 metres from a motorway and those living 300 metres away, or between those living 300 and 800 metres away. This 3.5 unit reduction is similar in magnitude to that observed in a clinical population experiencing reduced overall quality of life (3.3 units) [25]. In a general population, it is comparable to the difference between those not completing high school and tertiary graduates (4.2 units), or between those with and without a physical chronic condition (2.0 units) [25].

Our findings are consistent with previous cross-sectional studies linking traffic noise disturbance with lower wellbeing [13, 14]. In particular, one study found that mental wellbeing assessed using SF-36 was 4.2 units lower in those experiencing traffic noise disturbance than those not [13]. However, our findings are inconsistent with evaluations of other types of urban regeneration initiatives in the UK, which have found either no change [32] or modest improvements [8, 9] in wellbeing. A recent study of neighborhood demolition and housing improvement (also in Glasgow) found a significant increase in mental wellbeing in participants receiving housing improvements relative to controls, measured using SF-12 (2.4, 95% CI 0.0 to 4.8) [8].

### Implications for policy and practice

There is currently little public health evidence to guide policy decisions about investing in expensive urban regeneration projects. We found negative impacts of a new motorway on wellbeing. However, more time may be necessary for some benefits, such as economic revival (which we have not assessed directly), to be fully realised and impact on wellbeing. Those with chronic conditions living near an existing motorway experienced the greatest adverse effects on wellbeing, which may entrench existing health inequalities. From a social justice perspective, there did not appear to be a fair distribution of benefits and harms for those living near a motorway, particularly as approximately half of our sample did not own a car. Previous work on the socio-spatial patterning of busy roads and industrial sites indicates these are disproportionately located near deprived neighbourhoods [33, 34].

While transport policy in Scotland and other countries highlights the need to promote active travel and public transport on health and sustainability grounds [35], urban design continues to prioritise car use despite the adverse health effects associated with a car-dominant transport system [36]. This study will help inform future policy in the UK and further afield.

### Implications for research

Several issues may be of interest to researchers. Firstly, while delimiting area-based exposures in natural experimental research is relatively straightforward [37], in this study individual proximity to a motorway appeared to be a more meaningful exposure. This seems intuitive given that some of the hypothesised contributors to poor wellbeing, including visual intrusion and traffic noise, are restricted to those in close proximity. Graded proximity exposures have been employed in other recent natural experimental studies [31, 38]. In future studies, the optimal definition of exposure will depend on the particular combination of intervention, study design and outcome.

Secondly, the longitudinal and repeat cross-sectional analyses did not fully corroborate. At baseline, the cohort was significantly wealthier and had higher mental wellbeing on average than the rest of the T1 sample. It is therefore plausible that their response to the intervention differed from that of the repeat cross-sectional sample. The longitudinal analysis examined within-participant change over time and provided the greatest support for causal inference, but was limited by the smaller sample size. The repeat cross-sectional analysis examined population-level shifts over time, bolstering the sample size but providing a lower level of confidence for causal inference at individual level. It is likely that the intervention operated differently at the individual and population levels, reflecting the differences we found. This will be explored further in complementary quantitative mediation analyses and qualitative research.

Finally, in natural experimental research, replication is unlikely to involve multiple studies of the same intervention–rather, multiple studies between which researchers can synthesise the effects of altering the same general characteristics of the environment in different contexts. The cumulation of this work will allow researchers to make more generalisable causal statements about the effects of environmental change [18, 22].

### Conclusions

Living near to a new motorway appeared to worsen residents’ wellbeing. In an area with an existing motorway, negative impacts were concentrated in those with chronic conditions, which may exacerbate health inequalities and contribute to poorer health. Health impacts of this type of urban regeneration intervention should be considered in future policy and planning.

## Supporting information

S1 Appendix Study survey. (PDF)
