# Supplementary material for: Effects of living near an urban motorway on the wellbeing of local residents in deprived areas: Natural experimental study
Source: PLoS One. 2017 Apr 5;12(4):e0174882. doi: 10.1371/journal.pone.0174882 (PMC5381791; doi:10.1371/journal.pone.0174882)

Resp_ID

Bar code

[Name]
[Address]

[Date]

Dear [Name]

Traffic and Health in Glasgow

You may remember taking part in a research study on traffic and health in your local area in 2005. You also gave us permission to contact you again in the future to ask you to take part in a follow-up study. We are now conducting this follow-up study, and I am writing to invite you to participate. The study is being run by the MRC/CSO Social and Public Health Sciences Unit, University of Glasgow.

Before you decide whether to participate, it is important for you to understand why the research is being done and what it will involve. Please take time to read the following information carefully and discuss it with others if you wish. Take time to decide whether or not you wish to take part. Thank you for reading this letter.

What is the purpose of the study?
The conditions of city life are important for people's health. We want to find out how the local environment affects how people feel, how they travel around, and their general health. We are particularly interested in roads, traffic and transport and how these affect the quality of life in local areas. There may have been some changes in your area since 2005. We are interested in how things may have changed and how these changes have affected the people in your area. We will be surveying people who live in three different parts of the city.

Why have I been chosen?
In 2005 you filled out a survey on traffic and health in Glasgow. You also returned a consent form giving us permission to contact you again. We are now conducting a follow-up study and hope that you will consider giving us more information. We will be contacting about 1000 people from the 2005 survey, and also giving the survey to some new people. We hope that in total 3000 people will take part.

Do I have to take part?
It is up to you to decide whether or not to take part in this follow-up study. The survey is similar to the one you filled out in 2005.

What will happen to me if I take part?
If you return the survey, we will analyse this information and link it to the information you gave us in 2005. We will also combine it with the information from other people's surveys.

In addition, we hope you will consider filling in an optional **consent form** with your current contact details. If you return this form, then:

(a) During the next year, you will have a chance of being invited to talk about the topic in more detail in a one-to-one discussion. If you are chosen for this, we will send you a separate letter nearer the time.

AND

(b) You will have a chance to participate in an in-depth study in which your physical activity will be measured with a small device. Again, we will send you a separate letter about this nearer the time.

We are not asking you to agree to take part in either the one-to-one discussion or the physical activity measurement study now. We are only asking for permission to contact you again later to invite you to take part. Full information about these extra studies will be provided when we contact you. If you do decide to take part in either of these extra studies, you will still be free to withdraw at any time and without giving a reason.

What do I have to do now?
We would like you to fill in the survey. It will probably take about 30 minutes to complete. You have been provided with two **freepost** envelopes. Please send the survey back in the large envelope. If you choose to fill in the consent form, please send this back to us in the small envelope (keep it separate from the survey to ensure that your answers are confidential). No stamp is needed.

What are the possible disadvantages of taking part?
There is no disadvantage to you except for the time you will need to spend on the survey. We will not give your contact details to anyone else.

What are the possible benefits of taking part?
The information that is collected during this study will give us a better understanding of the health effects of traffic and the environment. We hope this will help make sure that future transport policy takes account of people's health needs. You will receive no direct benefit from taking part in this study, except that if you return a completed survey you will receive a £5 gift voucher.

Will my taking part in this study be kept confidential?
All information which is collected about you during the course of the research will be kept strictly confidential. You will be identified by an ID number and any information about you will have your name and address removed so that you cannot be recognised from it. Your contact details will be stored by the MRC/CSO Social and Public Health Sciences Unit, University of Glasgow. Your survey information, which will have an ID number, will be stored by the MRC Epidemiology Unit, University of Cambridge. Your information will be stored for 30 years.

What will happen to the results of the research study?
We expect to publish the results of the study in the next few years. No-one will be able to identify you personally in any results that are published. If you send back a survey, we will send you a summary of the overall results.

Who is organising and funding the research?
The research is organised by the MRC/CSO Social and Public Health Sciences Unit at the University of Glasgow, and the MRC Epidemiology Unit at the University of Cambridge, in collaboration with the Glasgow Centre for Population Health, University of East Anglia and University of Edinburgh. It is funded by the National Institute for Health Research.

Who has reviewed the study?
This study has been approved by the University of Glasgow College of Social Sciences Research Ethics Committee.

Contact for further information
If you have any questions about filling in the survey, please phone the free helpline at the survey office, MRC/CSO Social and Public Health Sciences Unit, 0800 389 2129.

If you would like further information about the study, please phone the free help line or e-mail survadmin@sphsu.mrc.ac.uk. Also, if you have any concerns about the conduct of this study you can contact the College of Social Sciences Ethics Officer Dr Valentina Bold at valentina.bold@glasgow.ac.uk.

Yours sincerely

Dr David Ogilvie PhD FFPH

Approved by the University of Glasgow
College of Social Sciences Ethics Committee
Ref:400120077

University of Glasgow

UNIVERSITY OF CAMBRIDGE

Bar code

MRC Medical Research Council

CHIEF SCIENTIST OFFICE

CONFIDENTIAL

Traffic and Health in Glasgow

Survey

Who should complete this survey?

This survey should be completed by **the person to whom the envelope was addressed**.
Please do not pass it on to another person or household.

How should I complete this survey?

The survey is not long. It should take about 30 minutes to complete.

Please use a blue or black pen.

Some questions ask you to **tick** a box. Please tick the box that applies to you.

Example: Are you male or female? Male ☑ Female ☐

Other questions ask you to **write numbers** in a box

Example: What is your age? *Write in* [53] years

Don't worry if you make a **mistake** — just cross out the mistake and put in the correct answer.

Example: Do you have access to a bicycle? Yes ☒ No ☑

Approved by the University of Glasgow
College of Social Sciences Ethics Committee
Ref:400120077

Section 1: About you and your household

1.1 Are you male or female? *Tick one only* Male ☐ 1 Female ☐ 2

1.2 What is your age? *Write in* ☐ years

1.3 OTHER THAN YOURSELF, how many people live in your household?

We mean people who have your accommodation as their only or main residence
AND
who either share at least one meal a day with you
OR
share the living accommodation (living room or sitting room) with you.

Write in number
If none, write '0'

1.3.1 Children aged under 5 ☐

1.3.2 Children aged between 5 and 15 ☐

1.3.3 Adults aged 16 and over (do not include yourself) ☐

1.4 Does your household own or rent its accommodation?

Tick one only

Rents it from the council, a housing association, or a charity ☐ 1

Rents it from a private landlord or letting agency ☐ 2

Partly owns it and partly rents it (shared ownership) ☐ 3

Owns it (including buying with a mortgage) ☐ 4

Other ☐ 5

1.5 Which of these best describes your situation at present? Please answer for yourself, and for your spouse or partner if you have one who lives with you.

**Yourself**	1.5.1 *Tick one only*	**Your spouse/partner**	1.5.2 *Tick one only*
		Not living with a spouse or partner	☐ 11
Doing paid work full time	☐ 1	Doing paid work full time	☐ 1
Doing paid work part time	☐ 2	Doing paid work part time	☐ 2
Doing voluntary work	☐ 3	Doing voluntary work	☐ 3
On a government training scheme	☐ 4	On a government training scheme	☐ 4
Retired	☐ 5	Retired	☐ 5
Full time student	☐ 6	Full time student	☐ 6
Unemployed	☐ 7	Unemployed	☐ 7
Disabled, invalid or permanently sick	☐ 8	Disabled, invalid or permanently sick	☐ 8
Caring for home and family or dependants	☐ 9	Caring for home and family or dependants	☐ 9
Other	☐ 10	Other	☐ 10

1.6 Do you have access to a bicycle? *Tick one only* Yes ☐ 1 No ☐ 2

1.7 How many cars or vans are owned, or available for use, by members of your household?
Do not include motorcycles, scooters or mopeds.

Write in number. If none, write '0' ☐

Approved by the University of Glasgow
College of Social Sciences Ethics Committee
Ref:400120077

1.8.1 Please give the full address and postcode of your usual place of work, study or training.
If you have more than one place of work, study or training, please give the address of the place to which you travel most often.

Postal address [ ]

1.8.2 Postcode [ ][ ][ ][ ][ ][ ][ ]

OR

Do not work or study [ ] 1

1.9.1 How far do you have to travel to get to your usual place of work, study or training?
If you have more than one place of work, study or training, please answer for the place to which you travel most often.

Tick one only

Do not work or study	[ ] 1	
Usually work at home or from home	[ ] 2	
Less than one mile	[ ] 3	
One mile or more	[ ] 4	→ *Write in number of miles* **1.9.2** [ ]

1.10 Thinking about the cost of living as it affects you and your household, which of these best describes your situation at present?

Tick one only

Find it a strain to get by from week to week	[ ] 1
Have to be careful about money	[ ] 2
Able to manage without much difficulty	[ ] 3
Quite comfortably off	[ ] 4

Section 2: About your travel

In this section, we are interested in all the journeys you made **yesterday** (between 3 a.m. yesterday morning and 3 a.m. this morning).

Please list each journey you made yesterday to get from place to place. These might include, for example, going to work, going out to get lunch, coming home from work, going shopping, going to the doctor's, visiting friends, or escorting someone else (e.g. taking child to school). Please include time spent travelling on foot or by bike, even if this was part of a longer journey (e.g. by bus or train). But please **do not include** journeys you made as part of your job (e.g. as a bus driver), or walking or cycling purely for recreation or exercise (e.g. walking the dog).

*We have given you an example of **one** journey. This person walked for ten minutes to the bus stop, rode on the bus for 22 minutes, and then walked for five minutes to get to work (a total of 15 minutes walking). They did not count the time spent waiting for the bus.*

2.1 Did you make any journeys yesterday? *Tick one only* Yes ☐ 1 No ☐ 2 → *If **NO** please go to question **2.3** below*

2.2

How many MINUTES did you spend TRAVELLING by each mode of transport on this journey?
Do not count time spent waiting for buses, trains, etc.

	What was the purpose of the journey?	Bus or coach	Train or underground	Car, taxi or van	Motorcycle or moped	Bicycle	Walking	Other
Example	To work	22					15	
Journey 1								
Journey 2								
Journey 3								
Journey 4								
Journey 5								
Journey 6								
Journey 7								
Journey 8								
Journey 9								
Journey 10								
Journey 11								
Journey 12								

2.3 What day of the week was it yesterday? *Tick one only* Mon ☐ 1 Tue ☐ 2 Wed ☐ 3 Thu ☐ 4 Fri ☐ 5 Sat ☐ 6 Sun ☐ 7

2.4 Were you at home any time yesterday? *Tick one only* Yes ☐ 1 No ☐ 2

2.5 Was yesterday a normal working day for you? *Tick one only* Yes ☐ 1 No ☐ 2 Not applicable ☐ 3

Approved by the University of Glasgow
College of Social Sciences Ethics Committee
Ref:400120077

Section 3: About your physical activity

In this section, we are interested in finding out about the kinds of physical activities that people do as part of their everyday lives. The questions will ask you about the time you spent being physically active in the **past seven days**. Please answer each question even if you do not consider yourself to be an active person. Please think about the activities you do at work, around your home and garden, to get from place to place, and in your spare time for recreation, exercise or sport.

For each question, write the numbers in the boxes to the left OR tick the box to the right, as appropriate.

3.1 Think about all the **vigorous activities** that you did in the **past seven days**. Vigorous physical activities refer to activities that take hard physical effort and make you breathe much harder than normal. Think *only* about those physical activities that you did for at least ten minutes at a time.

7 days

During the PAST SEVEN DAYS, on how many days did you do vigorous physical activities like heavy lifting, digging, five-a-side football, aerobics, running, or fast cycling?

Write in ☐ days per week **OR** No vigorous activities *Tick* ☐ 1 → Please go to question **3.3**

3.2 **How much time did you spend doing VIGOROUS physical activities on average on each of those days?**

Write in ☐ hours and ☐ minutes per day **OR** Don't know/not sure *Tick* ☐ 1

3.3 Think about all the **moderate activities** that you did in the **past seven days**. Moderate activities refer to activities that take moderate physical effort and make you breathe somewhat harder than normal. Think *only* about those physical activities that you did for at least ten minutes at a time.

7 days

During the PAST SEVEN DAYS, on how many days did you do moderate physical activities like carrying light loads, vacuuming, gardening, dancing, leisurely swimming, or cycling at a regular pace? Do not include walking.

Write in ☐ days per week **OR** No moderate activities *Tick* ☐ 1 → Please go to question **3.5**

3.4 **How much time did you spend doing MODERATE physical activities on average on each of those days?**

Write in ☐ hours and ☐ minutes per day **OR** Don't know/not sure *Tick* ☐ 1

3.5 **During the PAST SEVEN DAYS, on how many days did you walk for at least ten minutes at a time?**

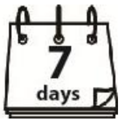

Write in ☐ days per week **OR** No walking *Tick* ☐ 1 → Please go to question **3.7**

3.6 **How much time did you spend WALKING on average on each of those days?**

Write in ☐ hours and ☐ minutes per day **OR** Don't know/not sure *Tick* ☐ 1

3.7 This last question is about the time you spent **sitting** on weekdays during the **past seven days**. Include time spent at work, at home, while doing course work and during leisure time. This may include time spent sitting at a desk, visiting friends, reading, or sitting or lying down to watch television.

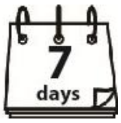

During the PAST SEVEN DAYS, how much time did you spend SITTING on average each weekday?

Write in ☐ hours and ☐ minutes per day **OR** Don't know/not sure *Tick* ☐ 1

Section 4: About your health

4.1 **Looking at the faces scale, which face shows best how you feel about your life as it is now?**

Tick one only

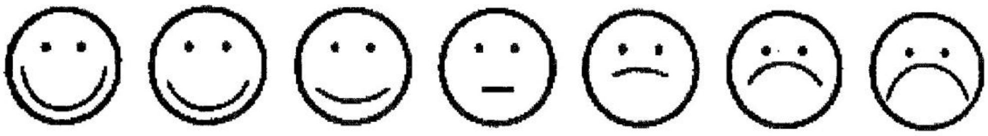

☐ 1 ☐ 2 ☐ 3 ☐ 4 ☐ 5 ☐ 6 ☐ 7

4.2 **Do you have any long-term illness, health problem or disability which limits your daily activities or the work you can do?** Include problems which are due to old age.

Tick one only Yes ☐ 1 No ☐ 2

Approved by the University of Glasgow
College of Social Sciences Ethics Committee
Ref:400120077

4.3 Do you have any difficulty walking for a quarter of a mile on the level?

Tick one only Yes ☐ 1 No ☐ 2

4.4 How tall are you? (with your shoes off)

Write in ☐ feet ☐ inches **OR** *Write in* ☐ centimetres

4.5 How much do you weigh? (in light indoor clothes)

Write in ☐ stones ☐ pounds **OR** *Write in* ☐ kilograms

The next section asks for your views about your health. This information will help keep track of how you feel and how well you are able to do your usual activities.

4.6 Overall, how would you rate your health during the PAST FOUR WEEKS?
Tick ONE box

4 weeks

Excellent	Very good	Good	Fair	Poor	Very poor
☐ 1	☐ 2	☐ 3	☐ 4	☐ 5	☐ 6

4.7 During the PAST FOUR WEEKS, how much did physical health problems limit your usual physical activities (such as walking or climbing stairs)? *Tick ONE box*

4 weeks

Not at all	Very little	Somewhat	Quite a lot	Could not do physical activities
☐ 1	☐ 2	☐ 3	☐ 4	☐ 5

4.8 During the PAST FOUR WEEKS, how much difficulty did you have doing your daily work, both at home and away from home, because of your physical health?
Tick ONE box

4 weeks

None at all	A little bit	Some	Quite a lot	Could not do daily work
☐ 1	☐ 2	☐ 3	☐ 4	☐ 5

4.9 How much BODILY pain have you had during the PAST FOUR WEEKS? *Tick ONE box*

None	Very mild	Mild	Moderate	Severe	Very severe
☐ 1	☐ 2	☐ 3	☐ 4	☐ 5	☐ 6

4.10 During the PAST FOUR WEEKS, how much energy did you have? *Tick ONE box*

Very much	Quite a lot	Some	A little	None
☐ 1	☐ 2	☐ 3	☐ 4	☐ 5

4.11 During the PAST FOUR WEEKS, how much did your physical health or emotional problems limit your usual social activities with family or friends? *Tick ONE box*

Not at all	Very little	Somewhat	Quite a lot	Could not do social activities
☐ 1	☐ 2	☐ 3	☐ 4	☐ 5

4.12 During the PAST FOUR WEEKS, how much have you been bothered by emotional problems (such as feeling anxious, depressed or irritable)? *Tick ONE box*

Not at all	Slightly	Moderately	Quite a lot	Extremely
☐ 1	☐ 2	☐ 3	☐ 4	☐ 5

4.13 During the PAST FOUR WEEKS, how much did personal or emotional problems keep you from doing your usual work, school or other daily activities? *Tick ONE box*

Not at all	Very little	Somewhat	Quite a lot	Could not do daily activities
☐ 1	☐ 2	☐ 3	☐ 4	☐ 5

SF-8™ 4-Week Recall Version — © 1999-2001 — QualityMetric, Inc. — All rights reserved

Below are some statements about feelings and thoughts. Please tick the box that best describes your experience of each over the **PAST TWO WEEKS**.

4.14 I've been feeling optimistic about the future *Tick ONE box*

None of the time	Rarely	Some of the time	Often	All of the time
☐ 1	☐ 2	☐ 3	☐ 4	☐ 5

4.15 I've been feeling useful *Tick ONE box*

None of the time	Rarely	Some of the time	Often	All of the time
☐ 1	☐ 2	☐ 3	☐ 4	☐ 5

4.16 I've been feeling relaxed *Tick ONE box*

None of the time	Rarely	Some of the time	Often	All of the time
☐ 1	☐ 2	☐ 3	☐ 4	☐ 5

4.17 I've been dealing with problems well *Tick ONE box*

None of the time	Rarely	Some of the time	Often	All of the time
☐ 1	☐ 2	☐ 3	☐ 4	☐ 5

4.18 I've been thinking clearly *Tick ONE box*

None of the time	Rarely	Some of the time	Often	All of the time
☐ 1	☐ 2	☐ 3	☐ 4	☐ 5

4.19 I've been feeling close to other people *Tick ONE box*

None of the time	Rarely	Some of the time	Often	All of the time
☐ 1	☐ 2	☐ 3	☐ 4	☐ 5

4.20 I've been able to make up my own mind about things *Tick ONE box*

None of the time	Rarely	Some of the time	Often	All of the time
☐ 1	☐ 2	☐ 3	☐ 4	☐ 5

Section 5: About your local area

This section asks for your views about your local area. Think of your local area as everywhere within a ten minute walk (about half a mile) from your home.

5.1 How long have you lived in your local area? If you have lived in this area previously and come back again, please just answer about the current period of time that you have lived in your local area.

10 minute walk

Write in ☐ Years ☐ Months

5.2 When did you move from your previous address to your current address? *Tick ONE box*

Jan ☐ 1	Feb ☐ 2	Mar ☐ 3	Apr ☐ 4	May ☐ 5	Jun ☐ 6
Jul ☐ 7	Aug ☐ 8	Sep ☐ 9	Oct ☐ 10	Nov ☐ 11	Dec ☐ 12

Year *Write in* ☐☐☐☐ **OR** I have always lived at my current address ☐ 1 → Please go to question **5.5**

5.3 Please give the full address and postcode of the last place you lived prior to your current address *Write in*

Postal address ☐

Postcode ☐☐☐☐☐☐☐

5.4 Please think back to the decision to move to your current address, and tick any considerations you thought were important at that time.

Tick all that apply

Cost	☐ 1	Access to shops and services	☐ 1
Access to recreational facilities	☐ 1	Visual characteristics of the local area	☐ 1
Access to public transport links (trains, buses)	☐ 1	Access to main roads	☐ 1
Length of commute for yourself	☐ 1	Length of commute for other adult(s) in the household	☐ 1
Aspects of the house (e.g. size, number of bedrooms, quality)	☐ 1	Characteristics of local residents	☐ 1
Local schools	☐ 1	Familiarity with local area	☐ 1
Child's commute to school	☐ 1	Near family or friends	☐ 1
Family/personal reasons (e.g. marriage, started living together, divorce, had children)	☐ 1	Other	☐ 1
Low crime	☐ 1	Moving was not my choice	☐ 1

Approved by the University of Glasgow
College of Social Sciences Ethics Committee
Ref:400120077

5.5 Looking at the faces scale, which face shows best how you feel about living in your local area?

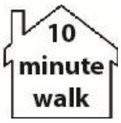

Tick one only

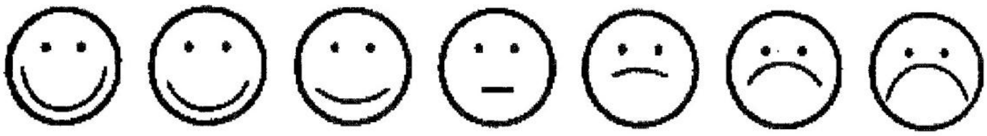

☐ 1 ☐ 2 ☐ 3 ☐ 4 ☐ 5 ☐ 6 ☐ 7

For each of the following statements about your local area, please tick one box to show how strongly you agree or disagree.

Tick one per row

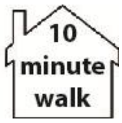

	**In my local area....**	**Strongly agree**	**Agree**	**Neither agree nor disagree**	**Disagree**	**Strongly disagree**
**5.6**	It is pleasant to walk	☐ 1	☐ 2	☐ 3	☐ 4	☐ 5
**5.7**	There is a lot of traffic noise	☐ 1	☐ 2	☐ 3	☐ 4	☐ 5
**5.8**	There is a park within walking distance	☐ 1	☐ 2	☐ 3	☐ 4	☐ 5
**5.9**	The roads are dangerous for cyclists	☐ 1	☐ 2	☐ 3	☐ 4	☐ 5
**5.10**	There is convenient public transport	☐ 1	☐ 2	☐ 3	☐ 4	☐ 5
**5.11**	People are likely to be attacked	☐ 1	☐ 2	☐ 3	☐ 4	☐ 5
**5.12**	There are convenient routes for cycling	☐ 1	☐ 2	☐ 3	☐ 4	☐ 5
**5.13**	There is little green space	☐ 1	☐ 2	☐ 3	☐ 4	☐ 5
**5.14**	It is safe to walk after dark	☐ 1	☐ 2	☐ 3	☐ 4	☐ 5
**5.15**	The nearest shops are too far to walk to	☐ 1	☐ 2	☐ 3	☐ 4	☐ 5
**5.16**	There is little traffic	☐ 1	☐ 2	☐ 3	☐ 4	☐ 5
**5.17**	There are no convenient routes for walking	☐ 1	☐ 2	☐ 3	☐ 4	☐ 5
**5.18**	It is safe to cross the road	☐ 1	☐ 2	☐ 3	☐ 4	☐ 5
**5.19**	The surroundings are unattractive	☐ 1	☐ 2	☐ 3	☐ 4	☐ 5

We are also interested in places you have walked or cycled to in your local area.
Please tick to show if you have walked or cycled to any of the following places in your **LOCAL AREA** in the **PAST SEVEN DAYS**. Remember that your local area is everywhere within a ten-minute walk (about half a mile) from your home. If you have walked **and** cycled to any of these local places in the past seven days, please tick both.

Tick all that apply

		Walked here in the past 7 days	Cycled here in the past 7 days	Did not walk or cycle here
5.20	Local shop (e.g. grocery shop, bakery, butcher)	☐ 1	☐ 1	☐ 1
5.21	Supermarket	☐ 1	☐ 1	☐ 1
5.22	Local services (e.g. bank, cash machine, post office, chemist, library)	☐ 1	☐ 1	☐ 1
5.23	Restaurant, cafe, pub or bar	☐ 1	☐ 1	☐ 1
5.24	Fast-food restaurant or takeaway	☐ 1	☐ 1	☐ 1
5.25	Bus stop, tram, train or underground station	☐ 1	☐ 1	☐ 1
5.26	Sport and leisure facility (e.g. swimming pool, sports field or fitness centre)	☐ 1	☐ 1	☐ 1
5.27	Open recreation area (park or other open space)	☐ 1	☐ 1	☐ 1
5.28	Family or friend's house	☐ 1	☐ 1	☐ 1
5.29	Work, school or training institute	☐ 1	☐ 1	☐ 1

5.30 Finally, we are interested in whether you walked or cycled in your local area **FOR RECREATION**, in the **PAST SEVEN DAYS** *Tick all that apply*

Walked for recreation in the past 7 days	Cycled for recreation in the past 7 days	Did not walk or cycle for recreation
☐ 1	☐ 1	☐ 1

Approved by the University of Glasgow
College of Social Sciences Ethics Committee
Ref:400120077

Section 6: About your neighbourhood

This section asks for your views about your neighbourhood. You can decide what area makes up your neighbourhood.

For each of the following statements, please tick one box to show how strongly you agree or disagree.

Tick one per row

		Strongly agree	Agree	Neither agree nor disagree	Disagree	Strongly disagree
6.1	People around here are willing to help their neighbours	☐ 1	☐ 2	☐ 3	☐ 4	☐ 5
6.2	This is close knit neighbourhood	☐ 1	☐ 2	☐ 3	☐ 4	☐ 5
6.3	People in this neighbourhood can be trusted	☐ 1	☐ 2	☐ 3	☐ 4	☐ 5
6.4	People in this neighbourhood generally get along with each other	☐ 1	☐ 2	☐ 3	☐ 4	☐ 5
6.5	People in this neighbourhood share the same values	☐ 1	☐ 2	☐ 3	☐ 4	☐ 5

Tick one per row

		Very likely	Likely	Neither likely nor unlikely	Unlikely	Very unlikely
6.6	If a group of neighbourhood children were skipping school and hanging out on a street corner, how likely is it that your neighbours would do something about it?	☐ 1	☐ 2	☐ 3	☐ 4	☐ 5
6.7	If some children were spray-painting on a local building, how likely is it that your neighbours would do something about it?	☐ 1	☐ 2	☐ 3	☐ 4	☐ 5
6.8	If there was a fight in front of your house and someone was being beaten or threatened, how likely is it that your neighbours would break it up?	☐ 1	☐ 2	☐ 3	☐ 4	☐ 5
6.9	If a child was showing disrespect to an adult, how likely is it that people in your neighbourhood would tell off or scold that child?	☐ 1	☐ 2	☐ 3	☐ 4	☐ 5

Section 7: Finally

Write in

**7.1** Please enter today's date.<br>We mean the date on which you filled in the survey.	[ ] *Date*	/ [ ] *Month*	/	2013

**Thank you for taking the time to complete this survey.
Your help is very important to our research, and very much appreciated.**

RETURNING THE SURVEY

Please remove both the information letter (at the front of this booklet) and the consent page (at the back of this booklet), then return the completed survey in the **larger FREEPOST envelope** provided.

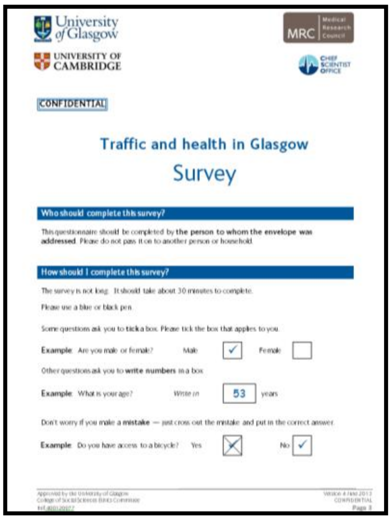

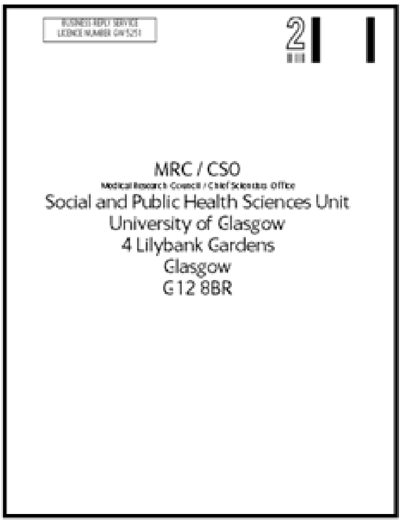

Resp_ID

Bar code

Traffic and Health in Glasgow
Work package 1

Lead Researcher: Dr David Ogilvie

Consent form for follow-up contact from the research team

Please initial **EACH** box

I confirm that I have read and understood the information letter for this study (version 1, dated June 2013).	☐
I give permission for the research team to contact me in the future, using the information I have supplied overleaf, to invite me to take part in extra studies relating to this survey. I note that I will be provided with full information about these extra studies if I am contacted again.	☐
I understand that my contact details will be held securely by the research team and will not be passed to anyone else.	☐
I understand that my participation in any future study or interview is voluntary and that I am free to withdraw at any time without giving any reason, without my legal rights being affected.	☐
I understand that if I want to withdraw this consent, I can do so at any time by writing to or telephoning the survey office at the MRC/CSO Social and Public Health Sciences Unit.	☐

Signed ______________________ Date ______________

Contact details

**First/given name(please print)**	
**Family name (please print)**	
**Address (please print)**	
**Postcode**	
**Telephone number (landline)**	
**Telephone number (mobile)**	
**Email**	

RETURNING THIS CONSENT PAGE

Please complete and detach this Consent page and return it in the **smaller FREEPOST envelope** provided.

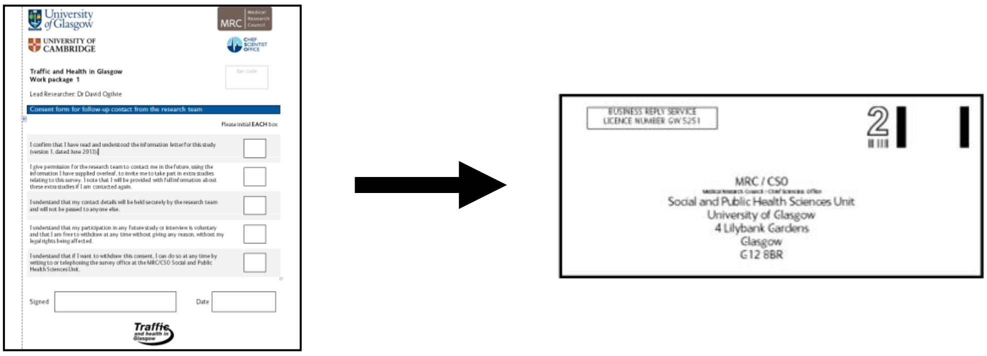

Supplement: S1 Appendix — (PDF) [file pone.0174882.s001.pdf]
